# Poorly differentiated is more significant than signet ring cell component for lymph node metastasis in mixed-type early gastric cancer: a retrospective study from a large-volume hospital

**DOI:** 10.1007/s00464-020-07532-5

**Published:** 2020-04-10

**Authors:** Jin-Nan Chen, Qi-Wen Wang, Qing-Wei Zhang, Zhao-Rong Tang, Xiao-Bo Li

**Affiliations:** 1 Shanghai Jiao Tong University School of Medicine Affiliated Renji Hospital, Shanghai, China; 2 Chongqing Hospital of Traditional Chinese Medicine, Chongqing, China

**Keywords:** Gastric cancer, Lymph node metastasis, Signet ring cell carcinoma

## Abstract

**Objective:**

The purpose of this study was to explore the role of different undifferentiated components in the lymph node metastasis (LNM) of early mixed gastric cancer.

**Methods:**

A total of 1596 patients with EGC who underwent gastrectomy were divided into four types: pure differentiated (PD), pure poorly differentiated (Poorly D), pure signet ring cell carcinoma (SRC), and mixed. Mixed type was classified into four subtypes: differentiated-predominant type mixed with poorly differentiated component (MD-P), poorly differentiated-predominant type mixed with differentiated component (MP-D), differentiated-predominant type mixed with SRC component (MD-S), and poorly differentiated-predominant type mixed with SRC component (MP-S). We analyzed the clinicopathological differences between all types and the rates of LNM of patients fulfilling endoscopic submucosal dissection (ESD) criteria.

**Results:**

LNM was more common in mixed (21.9%) than in PD (7.5%, *P* < 0.001) or SRC (11.3%, *P* < 0.001). When analyzed the subgroups of mixed type, LNM was more frequent in MD-P (15.4%) than in PD (7.5%, *P* = 0.003). LNM in MD-S (7.4%, *P* = 1.000) was not higher than in PD. MP-S (24.5%) showed a higher rate of LNM than SRC (11.3%, *P* < 0.001) rather than Poorly-D (22.7%, *P* = 0.681). For lesions satisfying ESD criteria, MD-S (0%, *P* = 1.000), and MD-P (5.9%, *P* = 0.12) did not have higher rates of LNM than PD (1.4%).

**Conclusion:**

The presence of poorly differentiated component but not SRC increases the LNM rate of mixed type. ESD is recommended for the treatment of MD-S and MD-P consistent with ESD criteria.

According to the latest global cancer statistics, gastric cancer is considered to be the fifth most common cancer in the world and the third leading cause of cancer-related deaths worldwide [1]. However, because of the progress of diagnostic methods, the detection rate of early gastric cancer (EGC) is gradually increasing [2]. Endoscopic submucosal dissection (ESD) is recommended as an effective treatment for EGC. It can effectively improve the prognosis of patients [3]. When determining whether patients with EGC should receive ESD treatment, the risk of lymph node metastasis (LNM) is the most important factor. LNM largely depends on tumor size, depth of invasion, and histology type [4]. In the Japanese Classification of Gastric Carcinoma (JCGC), gastric cancer is generally divided into differentiated and undifferentiated types. Undifferentiated gastric cancer mainly consists of poorly differentiated and signet ring cell types. Currently, the absolute indication of ESD is used for the treatment of differentiated gastric cancer confined to the mucosal layer without ulcer. It has been reported that undifferentiated EGC less than 2 cm in diameter has a low rate of LNM, so this is included in the expanded criteria [5, 6].

Mixed gastric cancer is defined as gastric cancer containing two or more different histological types. Mixed gastric cancer can be classified into differentiated-predominately mixed type (MD) and undifferentiated-predominately mixed type (MU) based on the main component [7]. Recently, several studies have shown that mixed gastric cancer is an independent risk factor for LNM in EGC [8–10]. Additionally, it has been reported that mixed gastric cancer is an LNM-predictive factor for submucosal EGC rather than for mucosal EGC [11–13]. In the presence of an undifferentiated component, the treatment of mixed gastric cancer needs to be cautious, while the guideline is still incomplete and controversial. Some studies have suggested that endoscopists need to judge the indications more careful when using ESD to treat mixed gastric cancer considering the low curative resection rate and high incidence of local recurrence of it [14–18]. However, Min et al. reported that ESD can be an effective therapy for differentiated-predominantly mixed gastric cancer that meets the absolute or expanded criteria [19]. In fact, undifferentiated carcinomas are mainly composed of Signet ring cell carcinoma (SRC) and poorly differentiated cancer (Poorly-D), and studies have shown that early SRC has a lower rate of LNM and a better survival rate [20]. However, relevant studies have not totally distinguished the undifferentiated components in mixed cancer. Thus, given the putative role of SRC in LNM, we focused on its role in LNM from mixed cancer. To achieve this goal, we divided the MD type into differentiated-predominant type mixed with SRC component (MD-S) and differentiated-predominant type mixed with poorly differentiated component (MD-P). We classified undifferentiated cancers into pure poorly differentiated cancer, pure signet ring cell cancer, poorly differentiated-predominant (MP) type mixed with differentiated component (MP-D), and poorly differentiated-predominant type mixed with SRC component (MP-S). All the groups were taken into the analysis.

The purpose of this study was to clarify the role of different undifferentiated components in the LNM of mixed gastric cancer. In addition, we investigated whether mixed gastric cancer satisfying the ESD criteria is suitable for ESD treatment.

## Materials and methods

### Patients

A total of 1596 surgically treated patients with EGC treated in Renji Hospital, Medical College of Shanghai Jiao Tong University, between July 2008 and December 2018 were enrolled in this retrospective study. Patients with incomplete pathological information, multiple gastric cancers, other malignancies, and invasion deeper than the submucosa were excluded. All data access was ethically approved.

Clinicopathologic features were obtained. These included age, gender, tumor size, location, macroscopic type, ulcer, depth of invasion, presence of LNM, N stage, and histology type.

### Definition and classification of mixed-type EGC

Histologically, all lesions were cut into three to five micron sections, and each section was stained with hematoxylin and eosin to observe the cell morphology and the presence of LNM. All the sections were retrospectively analyzed by two pathologists and divided into four types according to the 2010 World Health Organization (WHO) classification: PD type (consisting of well and moderately differentiated papillary adenocarcinoma and tubular adenocarcinoma), Poorly-D type (poorly differentiated adenocarcinoma), SRC type (signet ring cell carcinoma), and mixed gastric cancer (mixed) type. The mixed type was further classified into 4 subtypes according to the major component. These included MD-P (differentiated-predominant mixed-type with poorly differentiated component, which accounted for less than 50%), MP-D type (poorly differentiated-predominant mixed type with differentiated component, which accounted for less than 50%), MD-S type (differentiated-predominant mixed type with signet ring cell component, which accounted for less than 50%), and MP-S type (poorly differentiated-predominant mixed-type with signet ring cell component, which accounted for less than 50%). Three histological mixed type were not included in our research because of the small sample size. Macroscopic cancer was classified by the Japanese Gastric Cancer Association (JGCA) guidelines. These classes include the elevated types (I, IIa), the flat type (IIb), and the depressed types (IIc, III) [6]. Tumor location was identified as being in the upper third, middle third, lower third, or residual stomach. After lymph node dissection, the AJCC Cancer Staging Manual (eighth edition) was used to classify the N stage of the cancer.

### Statistical analysis

The *χ*^2^ test and multivariate analyses were performed to evaluate the effect of different mixed types on LNM. Multivariate analyses tested by logistic regression were used to compare groups. *P* < 0.05 was considered statistically significant. SPSS 23.0 for Windows was utilized for data analysis.

## Results

### Patients characteristics

Table 1 shows the clinical characteristics of all patients. The median age of the patients was 62 years (range 17–88), with 1069 males (67%) and 527 females (33%). According to the WHO classification, pathological types were divided into the following 4 groups: 723 (45.3%) PD, 181 (11.3%) Poorly-D, 203 (12.7%) SRC, and 359 (30.6%) mixed type. The overall rate of LNM was 14.1%, and the highest rate was found in patients with poorly differentiated gastric cancer (22.7%). The LNM rates of the mixed type were higher than those of the PD type (mixed versus PD, 21.9% versus 7.5%, respectively, *P* < 0.001) and the SRC type (mixed versus SRC, 21.9% versus 11.3%, respectively, *P* < 0.001), but were not different from those of the Poorly-D type (mixed versus Poorly-D, 21.9% versus 22.1%, respectively, *P* = 0.831).

**Table 1:** Clinical characteristic of early gastric cancer

	Total	PD	Poorly D	SRC	Mixed
	*N* = 1596	*N* = 723	*N* = 181	*N* = 203	*N* = 489
Age					
Median	62 (17–88)	62 (20–87)	59 (32–88)	59 (23–84)	62 (17–87)
Gender					
Male	1069 (67)	494 (68.3)	120 (66.3)	124 (61.1)	331 (66.7)
Female	527 (33)	229 (31.7)	61 (33.7)	79 (38.9)	158 (32.3)
Size					
(0, 10]	426 (26.7)	220 (30.4)	37 (20.4)	60 (29.6)	109 (22.3)
(10, 20]	626 (39.2)	287 (39.7)	71 (39.2)	80 (39.4)	188 (38.4)
(20, 30]	350 (22.6)	138 (19.1)	51 (28.2)	45 (22.2)	126 (25.8)
(30,)	184 (11.5)	78 (10.8)	22 (12.2)	18 (8.9)	66 (13.5)
Location					
Upper	204 (12.8)	135 (18.7)	14 (7.7)	7 (3.4)	48 (9.8)
Middle	399 (25.0)	166 (23)	46 (25.4)	58 (28.6)	129 (26.4)
Lower	979 (61.3)	418 (57.7)	118 (65.2)	137 (67.5)	306 (62.6)
Remnant	14 (0.9)	4 (0.6)	3 (1.7)	1 (0.5)	6 (1.2)
Depth					
M	813 (50.9)	402 (55.6)	65 (35.9)	150 (73.9)	196 (40.1)
Sm	783 (49.1)	321 (44.4)	116 (64.1)	53 (26.1)	293 (59.9)
Morphology					
Elevated	140 (8.8)	91 (12.6)	11 (6.1)	8 (3.9)	30 (6.1)
Flat	278 (17.4)	140 (19.4)	20 (11)	51 (25.1)	67 (13.7)
Depressed	1178 (73.8)	492(68)	150(82.9)	144(70.9)	392(80.2)
LNM					
Absent	1371 (85.9)	669 (92.5)	140 (77.3)	180 (88.7)	382 (78.1)
Present	225 (14.1)	54 (7.5)	41 (22.7)	23 (11.3)	107 (21.9)
N1	170 (75.6)	49 (90.7)	30 (73.2)	14 (60.9)	77 (72.0)
N2	55 (24.4)	5 (9.3)	11 (26.8)	9 (39.1)	30 (28.0)
Ulcer					
Absent	1431 (89.7)	660 (91.3)	155 (85.6)	181 (89.2)	435 (89.0)
Present	165 (10.3)	63 (8.7)	26 (14.4)	22 (10.8)	54 (11.0)

*PD* pure differentiated cancer, *Poorly D* poorly differentiated cancer, *SRC* signet ring cell cancer, *Mixed* mixed gastric cancer

### Comparisons of LNM among different histological types

To exclude other factors which may be involved in the LNM of EGC, age, gender, tumor size, tumor morphology, tumor location, ulcer, invasion depth, and pathological classification were integrated into a multivariate regression analysis. As shown in Table 2, tumor size, female sex [odds ratio (OR) 1.547; 95% confidence interval (CI) 1.131–2.116; *P* = 0.006], submucosal invasion (OR 3.836; 95% CI 2.682–5.486; *P* < 0.001), Poorly-D (OR 2.790; 95% CI 1.743–4.465, *P* < 0.001), mixed type (OR 2.945; 95% CI 2.039–4.253, *P* < 0.001), and SRC (OR 2.015; 95% CI 1.164–3.488, *P* = 0.012) were independent risk factors for LNM.

**Table 2:** Risk factors for lymph node metastasis in early gastric cancer

Variables	Multivariate		
	OR	95% CI	*P* value
Age	0.987	0.974–1.001	0.060
Female sex	1.547	1.131–2.116	0.006
Size ≤ 10 mm	Reference		
(10, 20]	1.857	1.161–2.970	0.010
(20, 30]	2.233	1.359–3.668	0.002
(30,)	3.021	1.730–5.277	0.000
Location at upper third	Reference		
Middle third	0.893	0.515–1.551	0.689
Lower third	1.023	0.622–1.682	0.930
Residual stomach	0.414	0.049–3.484	0.417
Morphology of elevated type	Reference		
Flat	0.878	0.442–1.745	0.710
Depressed	0.978	0.551–1.737	0.940
Ulcer (+)	1.014	0.634–1.622	0.954
Submucosal invasion	3.836	2.682–5.486	0.000
PD	Reference		
Poorly D	2.790	1.743–4.465	0.000
SRC	2.015	1.164–3.488	0.012
Mixed	2.945	2.039–4.253	0.000

*PD* pure differentiated cancer, *Poorly D* poorly differentiated cancer, *SRC* signet ring cell cancer, *Mixed* mixed gastric cancer

To investigate whether different types of mixed EGC had different risks for LNM, we divided them into 4 subgroups, including 130 (26.6%) MD-P, 144 (29.4%) MP-D, 188 (38.4%) MP-S, and 27 (5.5%) MD-S (Table 3). We compared the different mixed types with the pure histology type (Table 4).

When we compared the LNM rate of SRC with that of Poorly-D and MP-S, MP-S showed higher LNM (MP-S versus SRC, 24.5% versus 11.3%, respectively, *P* < 0.001). MP-S significantly predicted LNM (OR 1.923; 95% CI 1.075–3.441; *P* = 0.028). Although Poorly-D was more likely to have LNM (Poorly-D versus SRC, 22.7% versus 11.3%, respectively, *P* = 0.003), multivariate analysis showed that Poorly-D had no effective impact on LNM (OR 1.529; 95% CI 0.834–2.801, *P* = 0.17).

The LNM of MD-S was not higher than that of PD (MD-S versus PD, 7.4% versus 7.5%, respectively, *P* = 1.000), which was confirmed by multivariate analysis (OR 1.284; 95% CI 0.273–6.054; *P* = 0.752). Despite the fact that the Chi-square test showed that the LNM rate of SRC was not different from that of PD (SRC versus PD, 11.3% versus 7.5%, respectively, *P* = 0.078), multivariate analysis suggested SRC as an independent risk factor of LNM (OR 2.112; 95% CI 1.175–3.797; *P* = 0.013).

In addition, the MD-P type (MD-P versus PD, 15.4% versus 7.5%, respectively, *P* = 0.003) showed more frequent LNM than did the PD type (OR 1.904; 95% CI 1.052–3.446; *P* = 0.033). The same result (Poorly-D versus PD, 22.7% versus 7.5%, respectively, *P* < 0.001) was obtained in the Poorly-D type (OR 2.658; 95% CI 1.633–4.325; *P*<0.001). On the contrary, MP-D type (MP- versus Poorly-D, 27.1% versus 22.7%, respectively, *P* = 0.357) could not increase the incidence of LNM than that of the Poorly-D type (OR 1.265; 95% CI 0.734–2.182; *P* = 0.397).

We identified the differences between all the mixed types, namely MD-P, MP-D, MD-S, and MP-S. The LNMs of these types were 15.4%, 27.1%, 7.4%, and 24.5%, respectively. These results validated that MP-D (OR 2.270; 95% CI 1.190–4.328; *P* = 0.013) and MP-S (OR 2.037; 95% CI 1.074–3.862; *P* = 0.029) had more LNM than did MD-P, while MD-S showed no difference (OR 0.413; 95% CI 0.084–2.025; *P* = 0.276).

**Table 3:** Clinical characteristics of different subtypes of mixed early gastric cancer

	MD-P	MP-D	MD-S	MP-S
	*N* = 130	*N* = 144	*N* = 27	*N* = 188
Age				
Median	64 (17–86)	62 (29–85)	62 (48–82)	59 (24–87)
Gender				
Male	97 (74.6)	108 (75)	20 (74.1)	106 (56.4)
Female	33 (25.4)	36 (25)	7 (25.9)	82 (43.6)
Size				
(0, 10]	34 (26.2)	26 (18.1)	4 (14.8)	45 (23.9)
(10, 20]	44 (33.8)	58 (40.3)	8 (29.6)	78 (41.5)
(20, 30]	32 (24.6)	41 (28.5)	7 (25.9)	46 (24.5)
(30,)	20 (15.4)	19 (13.2)	8 (29.6)	19 (10.1)
Location				
Upper	26 (20.0)	11 (7.6)	0	11 (5.9)
Middle	31 (23.8)	32 (22.2)	6 (22.2)	60 (31.9)
Lower	70 (53.8)	100 (69.4)	21 (77.8)	115 (61.2)
Remnant	3 (2.3)	1 (0.7)	0	2 (1.1)
Depth				
M	47 (36.2)	45(31.3)	19(70.4)	85(45.2)
Sm	83 (63.8)	99(68.7)	8(29.6)	103(54.8)
Morphology				
Elevated	14 (10.8)	8 (5.6)	3 (11.1)	5 (2.7)
Flat	19 (14.6)	14 (9.7)	5 (18.5)	29 (15.4)
Depressed	97 (74.6)	122 (84.7)	19 (70.4)	154 (81.9)
LNM				
Absent	110 (84.6)	105 (72.9)	25 (92.6)	142 (75.5)
Present	20 (15.4)	39 (27.1)	2 (7.4)	46 (24.5)
N1	17 (85.0)	25 (64.1)	2 (100)	33 (71.7)
N2	3 (15.0)	14 (35.9)	0 (0)	13 (28.2)
Ulcer				
Absent	115 (88.5)	120 (83.3)	25 (92.6)	175 (93.1)
Present	15 (11.5)	23 (16.7)	2 (7.4)	13 (6.9)

*MD-P* differentiated-predominant mixed-type with poorly differentiated component accounted for less than 50%, *MP-D* poorly differentiated-predominant mixed-type with differentiated component accounted for less than 50%, *MD-S* differentiated-predominant mixed-type with signet ring cell component accounted for less than 50%, *MP-S* poorly differentiated-predominant mixed-type with signet ring cell component accounted for less than 50%

**Table 4:** Risk factors of lymph node metastasis in early gastric cancer compared between different subtypes of mixed gastric cancer

Variables	Multivariate		
	OR	95% CI	*P* value
SRC	Reference		
Poorly D	1.529	0.834–2.801	0.17
MP-S	1.923	1.075–3.441	0.028
PD	Reference		
SRC	2.112	1.175–3.797	0.013
MD-S	1.284	0.273–6.054	0.752
PD	Reference		
Poorly D	2.658	1.633–4.325	0.000
MD-P	1.904	1.052–3.446	0.033
Poorly D	Reference		
PD	0.375	0.232–0.606	0.000
MP-D	1.265	0.734–2.182	0 397
MD-P	Reference		
MD-S	0.413	0.084–2.025	0.276
MP-D	2.270	1.190–4.328	0.013
MP-S	2.037	1.074–3.862	0.029

*PD* pure differentiated cancer, *Poorly D* poorly differentiated cancer, *SRC* signet ring cell cancer, *MD-P* differentiated-predominant mixed-type with poorly differentiated component accounted for less than 50%, *MP-D* poorly differentiated-predominant mixed-type with differentiated component accounted for less than 50%, *MD-S* differentiated-predominant mixed-type with signet ring cell component accounted for less than 50%, *MP-S* poorly differentiated-predominant mixed-type with signet ring cell component accounted for less than 50%

### LNM rates in EGCs fulfilling the endoscopic resection criteria

We extracted the cases with tumor size less than 20 mm and with absence of ulcer and presence of mucosal invasion. As shown in Table 5, there was no difference in LNM rate between Poorly-D and PD (Poorly-D versus PD, 3.0% versus 1.4%, respectively, *P* = 1.000). When compared with PD type, LNM was more common in the Mixed type (Mixed versus PD, 11.8% versus 1.4%, respectively, *P* < 0.001) and the SRC type (SRC versus PD, 7.9% versus 1.4%, respectively, *P* < 0.003). However, such a distinction was not observed between the Mixed type and the Poorly-D type (Mixed versus Poorly-D, 11.8% versus 3.0%, respectively, *P* = 0.241) or the SRC type (Mixed versus SRC, 11.8% versus 7.9%, respectively, *P* = 0.333). Next, we analyzed different subgroups of mixed gastric cancer. The MD-P type (MD-P versus PD, 5.9% versus 1.4%, respectively, *P* = 0.12) and the MD-S type (MD-S versus PD, 0% versus 1.4%, respectively, *P* = 1.000) did not have any impact on the LNM. However, MP-D (MP-D versus PD, 13.3% versus 1.4%, respectively, *P* = 0.003) and MP-S (MP-S versus PD, 16.1% versus 1.4%, respectively, *P* < 0.001) did evidently increase the incidence of LNM.

**Table 5:** Rate of lymph node metastasis in patients meeting ESD treatment criteria

	Number	State of metastasis	
		Negative	Positive
Total	555	527 (95.0)	28 (5.0)
PD	294	290 (98.6)	4 (1.4)
Poorly D	33	32 (97.0)	1 (3.0)
SRC	101	93 (92.1)	8 (7.9)
Mixed	127	112 (88.2)	15 (11.8)
MD-P	34	32 (94.1)	2 (5.9)
MD-S	7	7 (100)	0 (0)
MP-D	30	26 (86.7)	4 (13.3)
MP-S	56	47 (83.9)	9 (16.1)

*PD* pure differentiated cancer, *Poorly D* poorly differentiated cancer, *SRC* signet ring cell cancer, *Mixed* mixed gastric cancer, *MD-P* differentiated-predominant mixed-type with poorly differentiated component accounted for less than 50%, *MP-D* poorly differentiated-predominant mixed-type with differentiated component accounted for less than 50%, *MD-S* differentiated-predominant mixed-type with signet ring cell component accounted for less than 50%, *MP-S* poorly differentiated-predominant mixed-type with signet ring cell component accounted for less than 50%

## Discussion

In our study, SRC was first extracted from the undifferentiated component in early mixed gastric cancer, and mixed cancer was further classified into four types: MD-P, MD-S, MP-D, and MP-S, to be taken into analysis. The results of our research showed that LNM was more likely to occur in the MD-P type than in the MD type, but not in the MD-S type. MP-D and MP-S did not increase the risk of LNM from the MP type. However, MP-S significantly increased the LNM of SRC. Next, we compared the patients who met ESD criteria and demonstrated that there was no difference in the LNM rate between the MD type and the PD type. On the contrary, MP-D and MP-S are more likely to cause LNM than PD.

Generally, undifferentiated cancer consists of poorly differentiated cancer and signet ring cell cancer. Many studies have reported that early SRC type tended to be confined to the mucosal layer and had a lower LNM rate and a higher 5-year survival rate than the non-SRC type [21–23]. On the contrary, the Poorly-D type is more likely to invade the submucosal layer, which may contribute to LNM. Several studies have shown that the MD type has higher LNM rates than the PD type, although there was no significant difference. However, these studies do not distinguish the undifferentiated components of the MD type [24, 25]. Considering these differences in histology heterogeneity, we hypothesized that the undifferentiated components of the MD type have different impacts on LNM and need to be evaluated and managed independently. The current study classified the MD type into an MD-S type and an MD-P type and revealed that the MD-P type has more aggressive behavior, as proven by the higher submucosal invasion rate and prevalence of LNM when compared with the PD type, while the MD-S type did not show these features. Meanwhile, our data also suggested that the MD-S type cannot be an effective predictor of LNM.

Studies on early SRC and the effect of the Poorly-D component on SRC remain controversial. A few studies have reported the LNM rate of early SRC ranges from 5 to 13.9%. Hu et al. reported a higher LNM rate of both the Poorly-D type and the MP-S type than SRC, although there was no statistical difference [17, 26–29]. Our study gave results consistent with this, showing that the LNM rate of SRC is 11.3%, lower than that of the MP-S type (21.9%) and the Poorly-D type (22.7%). In addition, to exclude factors other than the MP-S type, such as tumor size, submucosal invasion and ulceration, which may play a role in generating similar results, we performed a multivariate regression analysis and confirmed that MP-S was an independent risk factor compared to SRC, while Poorly D was despite its high LNM.

To see whether histologic predominance could affect the risk of LNM, we performed increased proportion of a poorly differentiated component in the mixed type, the LNM increased quickly, which validated that being poorly differentiated itself was an important factor that played a decisive role in the LNM of the mixed type, while SRC was not. However, this conclusion may remain controversial, as Seo’s study suggested that differences in composition of the mixed type do not cause differences in LNM [10]. Such inconsistencies may be due to the existence of a large amount of MP-D with submucosal invasion and small amounts of MD-S with mucosal invasion in our study.

At present, ESD is considered an effective method to treat early differentiated gastric cancer with diameter less than 2 cm limited to the mucosal layer without ulceration. Undifferentiated cancer with the same description is also included in the expanded criteria for ESD [6]. To investigate whether ESD can be used in the treatment of mixed gastric cancer, we screened out gastric cancer that meets the absolute or expanded criteria and found that the LNM rates from the mixed type and the SRC type were both higher than from the PD type, but not from the Poorly D type. We further analyzed the role of different subtypes of mixed gastric cancer and found that the differentiated-predominantly mixed type was not an independent risk factor for LNM for EGC limited in the mucosa when compared with PD. In contrast, the poorly differentiated-predominantly mixed type can effectively predict the rate of LNM in gastric cancer patients whose lesions are confined to the mucosal layer. The difference in the rate of LNM between surgical resected MD-P and MD-P meeting the ESD criteria may be attributed to the higher submucosal infiltration in the former, which is consistence with the previous study that poorly differentiated component is an independent risk factor for LNM in submucosal EGC [30]. Currently, a few studies have reported that a Poorly D component in the SRC type can significantly increase the rate of LNM and non-curative dissection of EGC. Even the MP-S type meets the ESD treatment criteria [15, 17]. In this study, although there was no statistical difference, the MP-S type that met the criteria were more prone to LNM than Poorly D and SRC. Additionally, considering the high rates of LNM of MP-S, surgical resection may be a better treatment for MP-S than ESD.

There are some limitations in this study. First, as this study is a retrospective study, there may be some data omissions and errors in the procedures of collecting data. However, all lesions were evaluated and pathologically diagnosed by two experienced pathologists to minimize errors. Second, only the specimens of EGC removed by surgery were included in this study, while the specimens of patients receiving ESD treatment were not included, which may lead to bias in selection. Third, we did not investigate the clinical outcome of patients. However, as for EGC, patients usually have a good prognosis. Fourth, we did not investigate the patient's history of *Helicobacter pylori*. As previously reported, *H. pylori* infection is not uncommon in undifferentiated cancer and may have an association with low LNM in SRC [31].

In conclusion, mixed gastric cancer is an independent risk factor for LNM in EGC. As for the undifferentiated components in differentiated-predominant EGC, these data suggest that the degree of malignancy of the tumor is significantly increased by poorly differentiated cancer when compared with SRC. In addition, for patients meeting the ESD treatment criteria, ESD treatment is recommended for differentiated-predominantly mixed cancer, while additional surgery is required for poorly differentiated-predominantly mixed cancer.
